# Indwelling catheter can increase postoperative urinary tract infection and may not be required in total joint arthroplasty: a meta-analysis of randomized controlled trial

**DOI:** 10.1186/s12891-018-2395-x

**Published:** 2019-01-05

**Authors:** Yimei Ma, Xiaoxi Lu

**Affiliations:** 1 Department of Pediatrics, West China Second University Hospital, Sichuan University, Chengdu, Sichuan 610041 People’s Republic of China; 2 Key Laboratory of Birth Defects and Related Diseases of Women and Children, Ministry of Education, Sichuan University, Chengdu, People’s Republic of China

**Keywords:** Total joint arthroplasty, Urinary catheterization, Urinary tract infection

## Abstract

**Background:**

The purpose of this study was to investigate whether patients undergoing total joint arthroplasty (TJA) require catheterization.

**Methods:**

PubMed, EMBASE, Web of Science, Cochrane Library and China National Knowledge Infrastructure were systematically searched. All randomized controlled trials (RCTs) receiving either a urinary catheterization or no urinary catheterization were included. Meta-analysis results were assessed by RevMan 5.3 software.

**Results:**

Seven independent RCTs were included, with a total sample size of 1533 patients, including 750 patients in the indwelling catheter group and 783 patients in the none-indwelling catheter group. Our pooled data analysis indicated that patients in the indwelling catheter group had a higher risk of urinary tract infection than patients in the none-indwelling catheter group (RR, 3.21; *P* = 0.0003). However, the meta-analysis indicated that there was no significant difference between the two groups in terms of urinary retention (RR, 0.67; *P* = 0.13), duration of the surgery (MD, − 0.37; *P* = 0.55), and length of hospital stay (MD, 0.15; *P* = 0.38).

**Conclusion:**

Based on the current evidence, this meta-analysis showed that urinary catheterization during TJA can increase the postoperative urinary tract infection, and it may not routinely be required for the patients undergoing TJA.

**Level of evidence:**

Level I, therapeutic study.

## Background

Total joint replacement (TJA) is considered to be one of the successful methods for the treatment of end-stage knee or hip disease [1–3]. However, postoperative urinary retention (POUR) occurs in TJA with an incidence between 0 and 75% [4], which is a common complication following TJA. Studies that show concerns that TKA and THA may lead to POUR [4, 5]. Prevention of POUR requires identification of patients with perioperative risk factors. At the same time, pharmacological strategies have been used to prevent or treat persistent POUR, but it has shown different effects and side effects in different types of surgery [6], therefore, the use of drugs remains controversial for preventing and treating POUR.

The use of an indwelling catheter can potentially increase postoperative urinary tract infections, and the duration of indwelling catheter group is the most important risk factor for urinary tract infections (UTI) [7]. UTI can lead to hematogenous bacteremia, seeding of the prosthesis implantation, and it eventually causes joint infection following TJA [8, 9]. An indwelling catheter is usually used for longer operations for checking the urinary output and guiding fluid resuscitation [10]. It was also widely used in the TJA performed under neuraxial anesthesia, which is considered to cause loss of the ability to sense bladder dilatation and neurogenic bladder problems [4].

n recent years, with the development of surgical techniques and anesthesia techniques, intraoperative blood loss has gradually decreased in TKA, thus making intraoperative fluid control less important. In addition, the clinical pathway of fast-track has achieved meaningful development in TJA [11–13]. Therefore, it also raises questions about the need for catheterization before surgery.

Based on the current clinical studies with urinary catheterization, we aimed to pool the results from the highest evidence-based (level I) studies to identify whether catheterization management is required in patients following primary TJA in terms of (1) urinary retention; (2) urinary tract infection; (3) duration of the surgery; and (4) length of hospital stay.

## Methods

The meta-analysis was based on the Cochrane Handbook for Systematic Reviews of Interventions [14] and was prepared in accordance with the PRISMA checklist guidelines. No ethical approval is required as it is a review of previously published articles and does not involve any processing of individual patient data.

### Search strategy

PubMed, Embase, Web of Science, the Cochrane Library and the China National Knowledge Infrastructure were systematically conducted up to June 2018. All of the comparative studies were involved in urinary atheterization for patients following TJA. The following keywords were used: “total knee arthroplasty”, “total knee replacement”, “total hip arthroplasty”, “total hip replacement”, “urinary catheterization”, “indwelling catheter”. There are no language or geographical restrictions.

### Inclusion criteria

The meta-analysis met the following criteria: PICOS (population, intervention, comparator, outcome, study design). (1) Population: patients were performed for primary TJA, including primary total knee arthroplasty and total hip arthroplasty; (2) Intervention: The intervention was urinary catheterization (indwelling catheter group); (3) Comparison: the comparator was none-indwelling catheter for TJA (none-indwelling catheter group); (4) Outcomes: the outcomes were the urinary retention, urinary tract infection, duration of the surgery and length of hospital stay. (5) Study design: the study design was performed by randomized controlled trials (RCTs);

### Assessment of methodological quality

Two reviewers assessed independently the methodological qualities of the study using the Cochrane Collaboration for Systematic Reviews. The seven items of sequence generation, allocation sequence concealment, blinding of participants and personnel, blinding of the outcome assessment, incomplete outcome data, selective reporting, and other biases were considered to be meaningful indicators. The overall methodological quality of each included study was measured as “low risk of bias”, “high risk of bias”, and “unclear risk of bias” [14].

### Data extraction and outcome measures

Full texts of studies that met the inclusion criteria were reviewed thoroughly. Two reviewers independently extracted the eligibility study results from the predefined data fields. The differences were resolved through discussion in order to reach a consensus. The following information was extracted, such as the first author, published date, age, number of participants, intervention method, anesthesia, criteria for urinary retention and outcome measures.

### Data synthesis

Statistical analyses were performed using RevMan 5 software (Version 5.3, the Cochrane Collaboration, UK). The continuous data, such as the duration of the surgery and length of hospital stay, the mean difference (MD) with 95% confidence interval (CI) were calculated. The dichotomous data, such as the urinary retention and urinary tract infection were calculated by risk ratio (RR) and 95% confidence interval (CI). Heterogeneity test was assessed using the chi-squared test and I^2^ statistic. If the chi-squared test > 0.05 or the I^2^ < 50%, the fixed effects model was used. A random-effects model was used if the chi-squared test < 0.05 or the I^2^ > 50%. Publication bias was assessed independently using funnel plots of the urinary tract infection.

## Results

### Search results

**Fig. 1:**
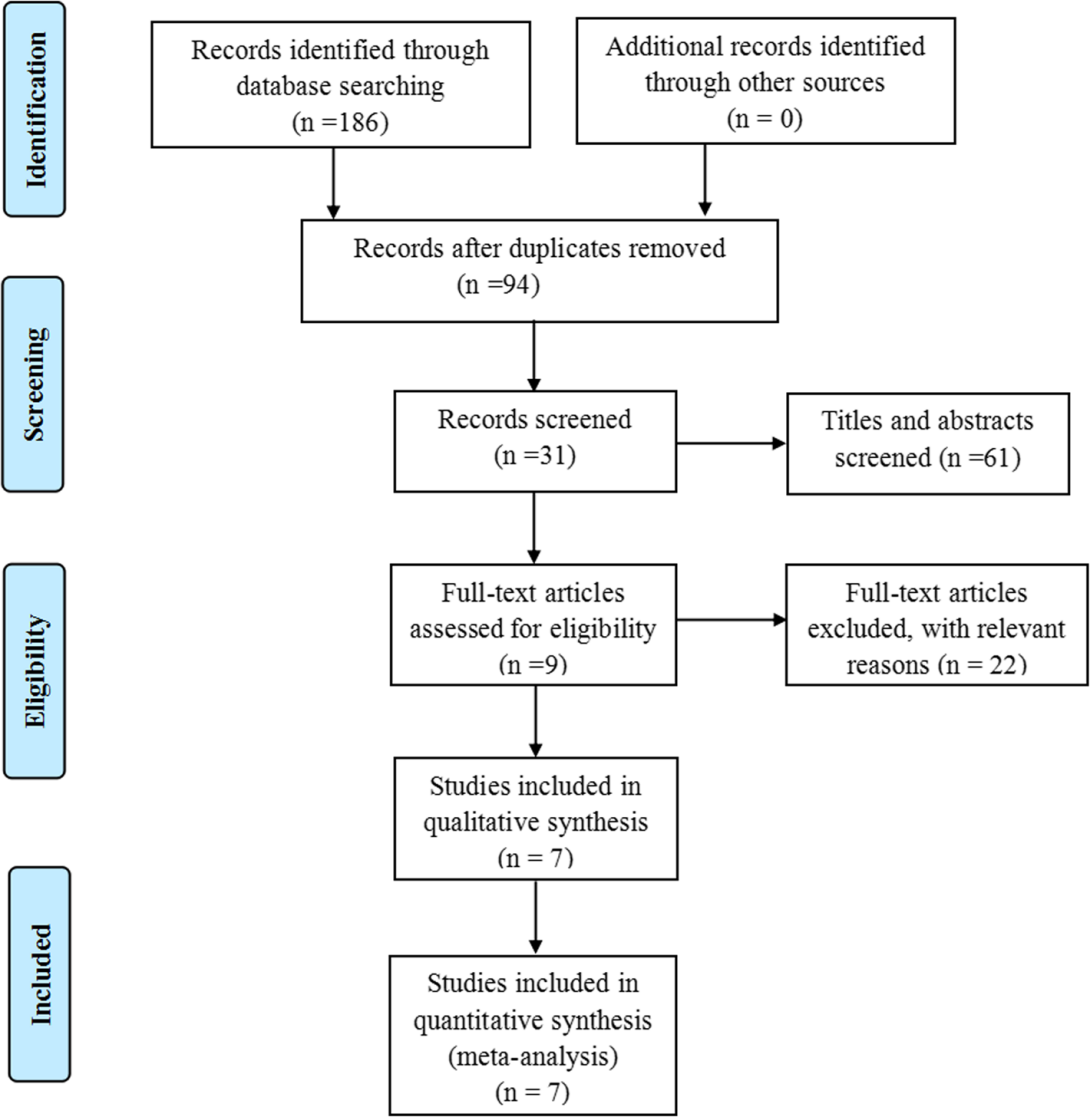
Preferred reporting items for systemic reviews and meta-analyses (PRISMA) flow diagram of literature selection

The flow chart of the study inclusion and exclusion was shown in Fig. 1. A total of 186 potentially relevant studies were identified through the search strategy, and 155 papers were read when excluding the duplicates and abstracts. According to the inclusion criteria, 7 RCTs [15–21] were finally included after reading the full text.

### Study characteristics

The meta-analysis included 750 patients in the indwelling catheter group and 783 patients in the none-indwelling catheter group. Sample sizes ranged from 30 to 346. All of the included studies were published between 2000 and 2018. The timing of the administration of indwelling catheter ranged from 8 h to 24 h after surgery. Although the criteria for urinary retention is different, most of the study is aimed at more than 400 ml. Five studies [15, 18–21] compared the indwelling catheter versus none-indwelling catheter in total knee arthroplasty, one study [16] compared these treatments in total knee arthroplasty and total hip arthroplasty, and one study [17] in total hip arthroplasty. Among them, 5 studies [16–21] have detailed the definition of UTI: In the study published by Van den Brand et al. [16], UTI is defined as a positive urine sediment for bacteria or white blood cells with a positive urine culture of 100,000 colonies. In the study published by Miller et al. [17], UTI was diagnosed from urine samples for culture and analysis from patients with symptoms suggesting urinary tract infections and patients undergoing postoperative urinary retention. In the study published by Huang et al. [18], UTI was defined as pyrexia or body temperature of 38 °C, urinary tract symptoms (dysuria, increased frequency of urination, urinary urgency, suprapubic pain and burning on micturition) and positive urine culture (> 107 bacterial colonies of microorganism forming units per liter). In the study published by Cai et al. [19], Urinary tract infection is defined as urine culture urinary colony count > 100,000 / ml, or symptomatic urine culture urinary colony count is > 100 / ml. In the study published by Luo et al. [20], UTI was defined as fever or body temperature of 38 °C, urinary tract symptoms and urine culture positive (bacteria count > 100,000 / mL). In the study published by Peng et al. [21], UTI was defined as having urinary tract irritation and urinary bacterial culture colony count > 100,000 / ml. Table 1 shows the baseline characteristics of all the included studies.

**Table 1:** Summary of study characteristics of the included study

		Age (y)		No of patients		Intervention method				
Author (date)	Surgery	CG	NCG	CG	NCG	CG	NCG	Anesthesia	Criteria for urinary retention	Outcomes
Lorio et al. 2000 [15]	TKA	67.8	66.8	306	346	Use a catheter for 24 h postoperatively	No catheter	General/Spinal /Epidural anesthesia	n.s.	2
Van den Brand et al. 2001 [16]	TKA and THA	68.6	68.2	46	53	Use a catheter for 48 h postoperatively	No catheter	General/Spinal anesthesia	n.s.	2
Miller et al. 2013 [17]	THA	60.1	58.7	107	93	Use a catheter for 8 h postoperatively	No catheter	Spinal anesthesia with bupivacaine	> 400 ml	1, 2
Huang et al. 2014 [18]	TKA	66.9	67.4	157	157	Use a catheter for 24 h postoperatively	No catheter	general anesthesia with saphenous nerve block	> 400 ml	1, 2, 3, 4
Cai et al. 2014 [19]	TKA	57.7	58.6	56	56	Use a catheter for 24 h postoperatively	No catheter	Intraveous combined inhaled anesthesia	> 400 ml	1, 2, 3, 4
Luo et al. 2017 [20]	TKA	68.9	67.2	30	30	Use a catheter for 24 h postoperatively	No catheter	General anesthesia	> 400 ml	1, 2, 3
Peng et al. 2018 [21]	TKA	66.8	67.4	48	48	Use a catheter for 24 h postoperatively	No catheter	Combined spinal and epidural anesthesia	> 500 ml	1, 2, 3

Abbreviation: *TKA* Total knee arthroplasty, *THA* Total hip arthroplasty, *CG* Catheter Group, *NCG* No-Catheter Group, *y* Year, *n.s*. not state, *1* Urinary retention, *2* Urinary tract infection, *3* Duration of the surgery, *4* Length of stay

### Risk of bias of the included studies

**Fig. 2:**
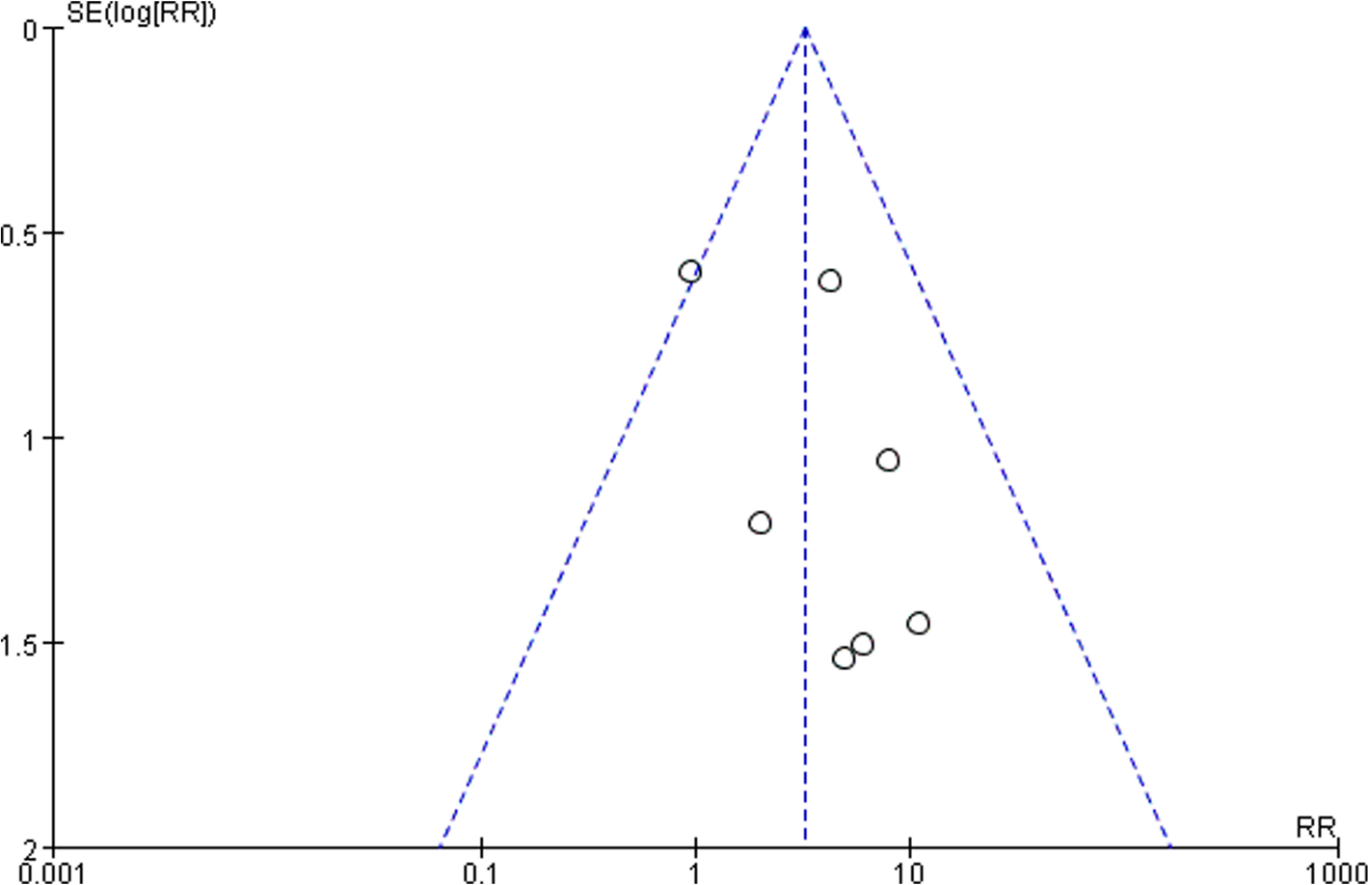
Publication bias of the urinary tract infection

Table 2 shows the risk of bias assessment of the 7 included studies. All of the studies were designed as RCTs and all studies reported incomplete outcome data, selective reporting, and other biases. The allocation concealment was stated four studies [17–20] and blinding were stated in 2 studies [17, 18]. The meta-analysis independently uses funnel plots of the urinary tract infection to assess publication bias; the plots were generally symmetrical and showed a lower publication bias (Fig. 2).

**Table 2:** Quality assessment of included 7 randomized controlled trials

Study	Level of evidence	Adequate Randomization method	Allocation concealment	Blinding of participants and personnel	Blinding of the outcome assessment	Incomplete outcome	Free of selective reporting	Free of other bias
Lorio et al. [15]	Level I	Yes	Unclear	Unclear	Unclear	Yes	Yes	Yes
Van den Brand et al. [16]	Level I	Yes	Unclear	Unclear	Unclear	Yes	Yes	Yes
Miller et al. [17]	Level I	Yes	Yes	Yes	Yes	Yes	Yes	Yes
Huang et al. [18]	Level I	Yes	Yes	Yes	Yes	Yes	Yes	Yes
Cai et al. [19]	Level I	Yes	Yes	Unclear	Unclear	Yes	Yes	Yes
Luo et al. [20]	Level I	Yes	Yes	Unclear	Unclear	Yes	Yes	Yes
Peng et al. [21]	Level I	Yes	Unclear	Unclear	Unclear	Yes	Yes	Yes

Abbreviations: *Yes* (low risk of bias), *Unclear* (unclear risk of bias)

### Meta-analysis of urinary retention

**Fig. 3:**
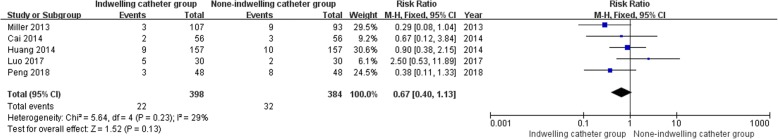
Meta-analysis of urinary retention

A total of 5 studies [17–21] reported relevant data on the urinary retention (398 and 384 patients in the indwelling catheter and none-indwelling catheter groups, respectively). The meta-analysis of urinary retention demonstrated that there was no significant difference between the two groups (risk ratio, 0.67; 95% CI, 0.40 to 1.13; *P* = 0.13). The pooled data did not show statistical heterogeneity, thus the fixed model was used (*P* = 0.23, I2 = 29%) (Fig. 3).

### Meta-analysis of urinary tract infection

**Fig. 4:**
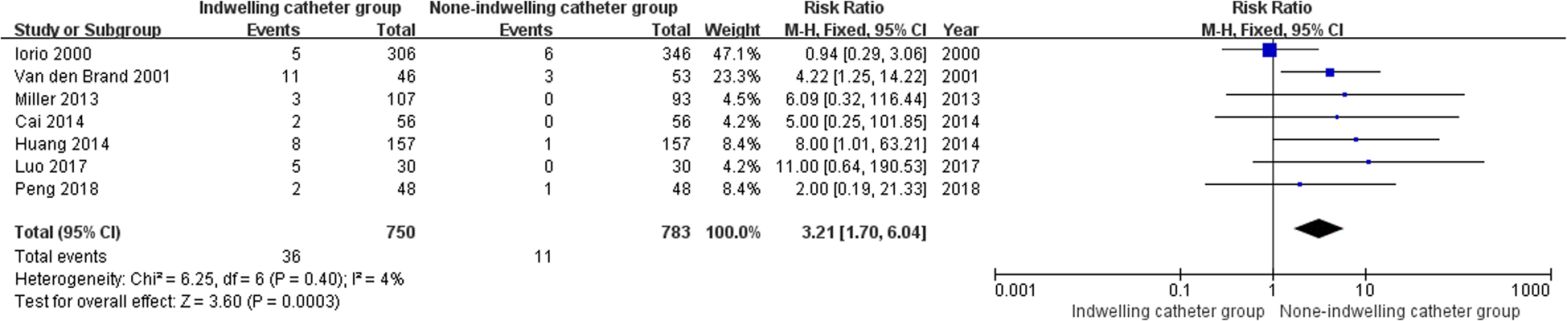
Meta-analysis of urinary tract infection

All studies [15–21] reported relevant data on the urinary tract infection (750 and 783 patients in the indwelling catheter and none-indwelling catheter groups, respectively). The meta-analysis of urinary tract infection demonstrated that patients in the indwelling catheter group had a higher risk of urinary tract infection than patients in the none-indwelling catheter (RR, 3.21; 95% CI, 1.70 to 6.04; *P* = 0.0003). The pooled data did not show statistical heterogeneity, thus the fixed model was used (*P* = 0.40, I2 = 4%) (Fig. 4).

### Meta-analysis of the duration of the surgery

**Fig. 5:**
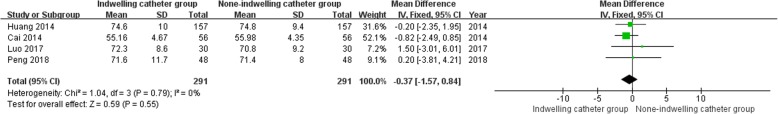
Meta-analysis of the duration of the surgery

A total of 4 studies [18–21] reported relevant data on the duration of the surgery (291 and 291 patients in the indwelling catheter and none-indwelling catheter groups, respectively). The meta-analysis of the duration of the surgery demonstrated that there was no significant difference between the two groups (MD, − 0.37; 95% CI, − 1.57 to 0.84; *P* = 0.55). The pooled data did not show statistical heterogeneity, thus the fixed model was used (*P* = 0.79, I2 = 0%) (Fig. 5).

### Meta-analysis of the length of hospital stay

**Fig. 6:**
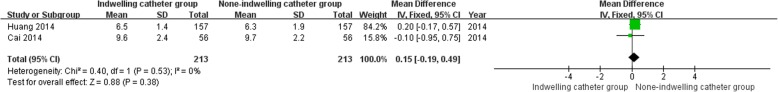
Meta-analysis of the length of hospital stay

A total of 2 studies [18, 19] reported relevant data on the length of hospital stay (213 and 213 patients in the indwelling catheter and none-indwelling catheter groups, respectively). The meta-analysis of the length of hospital stay demonstrated that there was no significant difference between the two groups (MD, 0.15; 95% CI, − 0.19 to 0.49; *P* = 0.38). The pooled data did not show statistical heterogeneity, thus the fixed model was used (*P* = 0.53, I2 = 0%) (Fig. 6).

## Discussion

There is still no standard protocol on whether or not to implement and apply indwelling catheter in TJA. Thus, we performed the meta-analysis comparing the efficacy and safety of urinary catheterization or not in total knee and hip arthroplasty. As far as we know, this is the first meta-analysis to report the above contents. The main finding in the current meta-analysis is that patients in the indwelling catheter group had a higher risk of urinary tract infection than patients in the none-indwelling catheter group. Additionally, there was no significant difference between the two groups in terms of urinary retention, duration of the surgery, and length of hospital stay. In total joint arthroplasty, preoperative catheterization is routinely used in lower limb arthroplasty to facilitate the monitoring of urine volume, guide fluid input, and prevent postoperative urinary retention. In recent years, the rapid development of fast-track clinical pathways and anesthesia technology, while reducing perioperative complications, the stable vital signs of patients are guaranteed smoothly. McDonald et al. [22] reported that the urinary catheterization rate of patients using fast-track guideline was significantly lower than that of the control group. In addition, with the rapid development of hemostasis and the continuous improvement of surgical techniques, the amount of blood lost during the primary unilateral TJA was generally controlled within 200 ml, and the complexity of monitoring urine volume during surgery was effectively reduced [22–24].

POUR is defined as having a full bladder but not being able to urinate on its own, which is a common complication after TJA. However, few studies have investigated the prevalence of this complication in TJA. At present, its quantitative criteria are still unclear, and it has become one of the important factors influencing clinical research [23]. Given the many factors affecting POUR, it is impossible to accurately exclude high-risk patients or control risk factors, therefore, there is still a risk of POUR after TJA without using an indwelling catheter. As previously reported, bladder volume over 500 ml for 4–24 h can result in bladder ischemia and decreased M receptor density, which can lead to persistent sexual dysfunction [25, 26]. In the Knight et al. [27] study, 119 patients undergoing TJA were randomized either to receive or not an indwelling catheter during surgery. The author reported a POUR rate of 35% in patients without an indwelling catheter than patients with a POUR rate of 19% in an indwelling catheter. The might explain why the POUR rate is higher than the other studies mentioned since most of the patients in this study received epidural catheter indwelling and indwelling analgesia within 48 h after the operation. In another study, Davis et al. [28] reported that the incidence of POUR following spinal anesthesia was significantly lower than that after epidural anesthesia (21.8% vs.46.7%) undergoing total hip arthroplasty.

The current meta-analysis is consistent with the results published in recent years. Huang et al. [18] performed 314 patients who underwent primary total knee arthroplasty and randomized them to receive either an indwelling catheter or not before the surgery. The result demonstrated that the prevalence of postoperative urinary retention (POUR) was quite low in both groups (5.7% vs 6.4%). Similarly, another RCT conducted by Miller et al. [17] also indicated that there was no significant difference between the indwelling catheter and none-indwelling catheter groups in terms of the prevalence of urinary retention and urinary tract infection in primary total hip arthroplasty. According to previous studies, Huang et al. [18] conducted a multivariate logistic regression for POUR identified age, male gender, ASA grade, duration of surgery, intraoperative intravenous fluid as the risk factors for increased urinary retention. Among them, age is the independent risk factor for POUR, and male gender and American society of anesthesiologists rating were considered as unchangeable development risk factors [23]. As for operative time and intraoperative intravenous infusion, with the development of surgical techniques and the importance of perioperative management, these risk factors can be well controlled through the use of tourniquet tranexamic acid and blood transfusion. These measures can significantly reduce intraoperative blood loss, and the requirement for such intraoperative fluid control is less important. In a prospective study, Karason et al. [29] found a correlation between POUR and bladder volume before anesthesia, suggesting that preoperative bladder residual urine volume greater than 100 ml was a risk factor for POUR, while bladder emptying before anesthesia was a protective factor.

The study also suggests that UTI are more likely to occur in patients with an indwelling catheter. UTI is considered to be an important factor in subsequent periprosthetic joint infection. As an invasive operation, indwelling catheter increases urethral injury, which is closely related to urinary tract and periprosthetic infection. Although some studies believe that the incidence of infection will not be increased within 24 h of the postoperative indwelling catheter, it can also cause discomfort, and hinder patients’ quick recovery [4, 15]. However, the accurate and timely diagnosis of UTI in TJA is very important to reduce potential infections. There are three potential reasons why TJA has a missed diagnosis of UTI: First, it should be suspected which patients have a high-risk factor for UTI in TJA, but there is little evidence of these factors. Second, the symptoms and signs of UTI are often non-specific and can easily cause confounding factors. Finally, obtaining uncontaminated samples can be challenging, and they can induce infection by invasive catheters and suprapubic suction sampling methods. As a result, the missed diagnosis of UTI can potentially increase the risk of UTI, delay the timing of treatment, and may eventually be customized around the prosthesis with blood flow, resulting in infection of the prosthesis. Therefore, the diagnosis of UTI requires a more accurate method in TJA.

The current study has several advantages: first, this is the first meta-analysis, and it includes high-quality RCT with strict inclusion criteria. Second, the relatively low incidence of POUR in patients in TJA encourages orthopedic surgeons to reconsider whether indwelling catheter should be used as a routine procedure. Third, the study found that the prevalence of UTI was significantly higher than that of patients using indwelling catheters, which potentially increased the risk of joint infection. There were also several limitations to the current study. First, the number of studies included and the sample size were relatively few in this meta-analysis. Second, there were no consistent criteria for urinary retention, although most of the definitions are that urine is greater than 400 ml. Third, the subgroup analysis was not performed according to the different anesthesia, the type of surgery and the time of indwelling catheter retention. Therefore, more high-quality articles are needed to confirm the above conclusions.

## Conclusions

Based on the current evidence, this meta-analysis showed that urinary catheterization during TJA, it can increase the postoperative urinary tract infection, may not be required for the patients undergoing TJA.

## Abbreviations

CI: Confidence interval
MD: Mean difference
POUR: Postoperative urinary retention
TJA: Total joint arthroplasty
UTI: Urinary tract infections

## References

[1] Kurtz SM, Ong KL, Lau E, Bozic KJ (2014). Impact of the economic downturn on total joint replacement demand in the United States: updated projections to 2021. J Bone Joint Surg Am.

[2] Dong CC, Dong SL, He FC. Comparison of Adductor Canal Block and Femoral Nerve Block for Postoperative Pain in Total Knee Arthroplasty. A Systematic Review and Meta-analysis. 10.1097/MD.0000000000002983 PMC4998367 27015172

[3] Kurtz S, Ong K, Lau E, Mowat F, Halpern M (2007). Projections of primary and revision hip and knee arthroplasty in the United States from 2005 to 2030. J Bone Joint Surg Am.

[4] Balderi T, Carii F (2010). Urinary retention after total hip and knee arthroplasty. Minerva Anestesiol.

[5] Griesdale DE, Neufeld J, Dhillon D, Joo J, Sandhu S, Swinton F, Choi PT (2011). Risk factors for urinary retention after hip or knee replacement: a cohort study. Can J Anaesth.

[6] Baldini G, Bagry H, Aprikian A, Carli F (2009). Postoperative urinary retention: anesthetic and perioperative considerations. Anesthesiology.

[7] Pulido L, Ghanem E, Joshi A, Purtill JJ, Parvizi J (2008). Periprosthetic joint infection: the incidence, timing, and predisposing factors. Clin Orthop Relat Res.

[8] Berbari EF, Hanssen AD, Duffy MC, Steckelberg JM, Ilstrup DM, Harmsen WS, Osmon DR (1998). Risk factors for prosthetic joint infection: case–control study. Clin Infect Dis.

[9] Ollivere BJ, Ellahee N, Logan K, Miller-Jones JC, Allen PW (2009). Asymptomatic urinary tract colonisation predisposes to superficial wound infection in elective orthopaedic surgery. Int Orthop.

[10] Wilde MH, Crean HF, McMahon JM, McDonald MV, Tang W, Brasch J, Fairbanks E, Shah S, Zhang F (2016). Testing a model of self-Management of Fluid Intake in community-residing long-term indwelling urinary catheter users. Nurs Res.

[11] Stowers MD, Manuopangai L, Hill AG, Gray JR, Coleman B, Munro JT (2016). Enhanced recovery after surgery in elective hip and knee arthroplasty reduces length of hospital stay. ANZ J Surg.

[12] Klapwijk LC, Mathijssen NM, Van Egmond JC, Verbeek BM, Vehmeijer SB (2017). The first 6 weeks of recovery after primary total hip arthroplasty with fast track. Acta Orthop.

[13] Kehlet H (2013). Fast-track hip and knee arthroplasty. Lancet.

[14] Higgins JP, Altman DG, Gotzsche PC, Juni P, Moher D, Oxman AD (2011). The Cochrane Collaboration's tool for assessing risk of bias in randomised trials. BMJ (Clinical research ed).

[15] Iorio R, Healy WL, Patch DA, Appleby D (2000). The role of bladder catheterization in total knee arthroplasty. Clin Orthop Relat Res.

[16] van den Brand IC, Castelein RM. Total joint arthroplasty and incidence of postoperative bacteriuria with an indwelling catheter or intermittent catheterization with one-dose antibiotic prophylaxis: a prospective randomized trial. J Arthroplast. 2001;16:85O-5. 10.1054/arth.2001.25547 11607900

[17] Miller AG, McKenzie J, Greenky M, Shaw E, Gandhi K, Hozack WJ, Parvizi J (2013). Spinal anesthesia: should everyone receive a urinary catheter?: a randomized, prospective study of patients undergoing total hip arthroplasty. J Bone Joint Surg Am.

[18] Huang Z, Ma J, Shen B, Pei F (2015). General anesthesia: to catheterize or not? A prospective randomized controlled study of patientsundergoing total knee arthroplasty. J Arthroplasty.

[19] Cai DF, Ma J, Huang ZY, Kang PD, Yang J, Shen B, Zhou ZK, Pei FX (2014). Safety and efficacy of non-indwelling urinary catheter in patients received intravenous combined inhaled anesthesia in total knee arthroplasty. A pilot studyInt J Orthop.

[20] Luo QP, Wang J, Yang J (2017). Safety and feasibility of preoperative non-indwelling catheter in primary unilateral total knee arthroplasty without tourniquet. West Chin Med J.

[21] Peng GF, Jin YN, Xu XF, Li JF, Wang QD, Kang L, Li FB (2018). Feasibility of unilateral total knee arthroplasty without urinary catheterization under combined spinal and epidural anesthesia. Int J Orthop.

[22] McDonald DA, Siegmeth R, Deakin AH, Kinninmonth AW, Scott NB (2012). An enhancedrecovery programme for primary total knee arthroplastyin the United Kingdom--follow up at one year. Knee.

[23] Bjerregaard LS, Bagi P, Kehlet H (2014). Postoperative urinary retention(POUR) in fast-track total hip and knee arthroplasty. Acta Orthop.

[24] Yang Y, Lv YM, Ding PJ, Li J, Ying-Ze Z (2015). The reduction in blood losswith intra-articular injection of tranexamic acid in unilateraltotal knee arthroplasty without operative drains: a randomizedcontrolled trial. Eur J Orthop Surg Traumatol.

[25] Balderi T, Mistraletti G, D'Angelo E, Carli F (2011). Incidence of postoperative urinary retention (POUR) after joint arthroplasty and management using ultrasound- guided bladder catheterization. Minerva Anestesiol.

[26] Kitada S, Wein AJ, Kato K, Levin RM (1989). Effect of acute completeobstruction on the rabbit urinary bladder. J Urol.

[27] Knight RM, Pellegrini VD (1996). Bladder management after total joint arthroplasty. J Arthroplast.

[28] Davis S, Erskine R, James MF (1992). A comparison of spinal and epidural anaesthesia for hip arthroplasty. Can J Anaesth.

[29] Karason S, Olafsson TA (2013). Avoiding bladder catheterisation in total knee arthroplasty: patient selection criteria and lowdose spinal anaesthesia. Acta Anaesthesiol Scand.

